# Orofacial findings associated with obstructive sleep apnea in a group of Saudi Children

**DOI:** 10.12669/pjms.312.6718

**Published:** 2015

**Authors:** Nouf S. AlHammad, Lujain A. Hakeem, Fouad S. Salama

**Affiliations:** 1 Nouf S. Al-Hammad, BDS, MS. College of Dentistry, King Saud University, Riyadh, Saudi Arabia; 2 Lujain A. Hakeem, BDS. College of Dentistry, King Saud University, Riyadh, Saudi Arabia; 3 Fouad S. Salama, BDS, MS. College of Dentistry, King Saud University, Riyadh, Saudi Arabia

**Keywords:** Children, Orofacial Variations, Sleep Apnea

## Abstract

**Objectives:**

To evaluate orofacial and occlusion findings associated with obstructive sleep apnea (OSA) in a group of Saudi children.

**Methods:**

The sample consisted of 30 OSA patients, and 30 age and gender matched, healthy control. The following facial and occlusal features were evaluated: frontal view, facial profile, mandibular angle, tongue size, dental midline to facial midline, upper to lower dental midline, overjet, overbite, anterior open bite, cross bite, scissors bite, palatal vault, maxillary and mandibular arch crowding and spacing, molar and canine relationship.

**Results:**

Participants age ranged from 3 to 8 years. Study group had steeper mandibular angle, deeper palatal vault, and less spaced upper and lower arches. There was no statistically significant difference between the two examined groups regarding facial morphology, facial profile, midline, anterior openbite, tongue size, posterior crossbite, overjet or molar relationship.

**Conclusion:**

OSA children have a relatively different orofacial morphology compared with control children. OSA subjects had deeper palatal vault, steeper mandibular plane angle and less spaced upper and lower arches compared to control.

## INTRODUCTION

There are variety of disorders related to sleep in children that ranges from simple, occasional snoring with no accompanying complications, through increased blockage of the upper airways to obstructive sleep apnea (OSA) where respiratory difficulties accompanied by hypoxemia, hypercapnia, and structural sleep difficulties.^1^,^2^ OSA is a common and serious cause of metabolic, cardiovascular, and neurocognitive morbidity in children.^3^ This disorder is characterized by repeated episodes of prolonged upper airway obstruction and/or intermittent complete obstruction that disrupts normal sleep patterns.^3^-^5^ OSA is a widespread disorder with a prevalence of 4 to 9 percent in the world’s population and it occurs in all age groups, but weight, age, race, and gender all influence its expression.^6^ OSA is as common as asthma and in its severe form can cause fatal cardiovascular damage.^6^

Little attention has been paid to the possible effects of sleep-related breathing disorders on the developing dentition.^2^ A review of the literature yielded that most of the conducted studies from different parts of the world indicated that children with sleep related breathing disorders have differences in dental arch dimensions and dentition compared with healthy children. These differences include adenoidal face, narrower maxilla, deeper palatal height, retrusive mandible, deficient chin, and long lower face, cross bites, increased overjet, and oral breathing.^1^-^3^,^5^,^7^-^12^ A study contributed those differences to the long-term changes in the position of the head, mandible, and tongue in order to maintain airway adequacy during sleep.^2^

Therefore, dentists should be aware of the many separate anatomic factors that contribute to these disorders and identify patients at risk in dental office to possibly correcting them early.^6^,^13^,^14^ As there are no previous studies on the association between orofacial findings and OSA for children in Saudi Arabia, our aim was to evaluate orofacial and occlusion findings associated with OSA in a group of Saudi children in Riyadh city.

## METHODS

The study protocol and consent form was approved by the Research and Ethical Committee of Human Studies at King Saud University, College of Dentistry Research Center. A single examiner (LH) who was trained and calibrated (kappa=0.78) with a senior faculty in recording occlusal features carried out all the examination procedures. The intra-examiner reliability was established by re-examination of 10 healthy children in 2 different visits with one week a part (Kappa=0.88).

The sample consisted of 30 OSA patients who were recruited from Ear-Throat and Nose (ENT) Clinic of King Abdul-Aziz University Hospital (KAUH) and Al Habeeb Private Medical Center, in Riyadh, Saudi Arabia, after being diagnosed by one Pediatric Otorhinolaryngologist and scheduled for surgical intervention. A control group of 30 healthy, none OSA children, randomly selected from patients attending College of Dentistry, matched for age and gender was similarly examined.

The following facial and occlusal features were evaluated according to the criteria, described by Huynh et al.11, by placing the child in the physiologic natural head position: Frontal view of the facial third analysis, facial profile, mandibular angle, tongue size, Facial medline to dental midline, upper to lower dental midline, overjet, overbite, anterior open bite, cross bite, scissors bite, palatal vault, maxillary and mandibular arch crowding and spacing, molar and canine relationships.

The collected data were recorded on a special form and the Statistical Package for Social Science (SPSS V.16) was utilized for statistical computation. Data analysis included frequency distribution; Chi-square, proportional t-test and independent t-test were used according to the nature of the variables studied, with the level of significance set at P≤0.05.

## RESULTS

The age of the children in each group ranged between 3-8 years, with a mean (±SD) age of 4.3 ±1.57 years. Males and females were equally distributed (50%) in both groups. The majority of the subjects in each group (80%) had primary dentition, while the remaining (20%) had mixed dentition. Analysis of data by age or dentition type was not done as the children were not evenly distributed by those two variables. There was no statistically significant difference in any of the variables tested between male and female children in both groups.

### Facial and occlusal features

For morphologic characteristics in the vertical plane: most subjects had normocephalic facial morphology in both control and study groups (73.3%) and (60%) respectrively. Dolicofacial, or long facial morphology, was observed in (10%) of the control group compared to (16.7%) in the study group. More than half (56.7%) of the control group had convex profile compared to (46.7%) in the study group. Only few subjects {2 (6.6%) in the study and 1 (3.3%) in the control group} had concave facial profile. There was no statistically significant difference (P>0.05) between the two examined groups in facial morphology or facial profile. Two cases (one in the control and one in the study group) were not included in the anterior teeth relation as their lower anterior teeth were missing.

For the facial midline with dental midline a discrepancy was found in only one case of the control group. Regarding the upper and lower dental midline, a deviation was noticed in (30%) of the control group compared to (16.7%) of the study group cases. Only one upper midline deviation was recorded in one of the controlled cases with no statistically significant difference between the two groups.

Only few cases of the study {4 (13.8%)} and control {2 (6.9%)} groups were recorded as having an anterior openbite with p>0.05. The remaining cases were evaluated for the amount of overbite. In control group out of the 27 control cases, 23 (85.2%) had overbite ranging from 0-50% and 4 (14.8%) had 51-100%. The corresponding numbers for the 25 cases in study group were 18(72%) and 7(28%) respectively, with no statistically significant difference recorded.

Tables I-V shows distribution of mandibular plane angle, palatal Vault (transverse plane), maxillary and mandibular arch crowding and spacing, molar and canine relations respectively between the groups. Majority of the cases in both control (86.7%) and study (96.7%) groups had normal tongue size with no statistically significant difference (p>0.05). Scissor bite and anterior crossbite were not registered in any of the examined cases, while posterior crossbite was found to be higher in the study group (23.3%) compared to the control (6.7%) with no statistically significant difference (p=0.063). None of the control group children had bilateral posterior crossbite which was recorded in two (6.6%) of the study group children.

**Table-I:** Mandibular Angle.

Mandibular Angle				
Group	N	Flat	Normal	Steep
Control	30	1 (3.3%)	27 (90%)	2 (6.7%)
Study	30	0 0%)	19 (63.3%)	11 (36.7%)
P value		0.3	0.01*	0.00*

* Significant

**Table-II:** Palatal Vault.

Palatal Vault			
Group	N	Deep	Round
Control	30	6 (20.0%)	24 (80.0%)
Study	30	18 (60.0%)	12 (40%)
P value		0.00*	0.00*

* Significant

**Table-III:** Maxillary and Mandibular arch spacing and crowding.

		Maxillary Arch			Mandibular Arch		
Group	N	Normal	Crowding	Spacing	Normal	Crowding	Spacing
Control	30	18(60%)	2(6.7%)	10(33.3%)	19(63.3%)	3(10.0%)	8(26.7%)
Study	30	27(90%)	2(6.7%)	1(3.3%)	25(83.3%)	5(16.7%)	0(0%)
P value		0.004*	1	0.001*	0.07*	0.44	0.001*

* Significant

**Table-IV:** Molar Relation.

Molar Relation						
Group	N	(R_L)Flush Terminal	(R_L)Mesial Step	(R_L)Distal Step	(R_L)Class I	Cross Arch
Control	30	2(6.7%)	21(70%)	1(3.3%)	3(10%)	3(10%)
Study	29*	5(17.2%)	18(62.1%)	0(0%)	4(13.8%)	2(6.9%)
P value		0.20	0.51	0.3	0.65	0.66

* One of the study group cases had bilateral missing lower posterior teeth.

**Table-V:** Canine Relation.

Canine Relation						
Group	N	(R_L)End to End	(R_L)Class I	(R_L)Class II	(R_L)Class III	Cross Arch
Control	30	1 (3.3%)	25 (83.3%)	1 (3.3%)	0 (0%)	3 (10%)
Study	30	5 16.7%)	18 (60%)	0 (0%)	5 (16.7%)	2 (6.6%)
P value		0.07	0.03*	0.30	0.014*	0.64

* Significant

In the sagittal or anteroposterior plane: an increased overjet (≥5mm) was recorded in few of the cases in both control and study groups (6.9% & 13.8%) respectively, with no statistically significant difference found.

## DISCUSSION

The present study has provided important baseline information on orofacial findings associated with OSA in a group of Saudi children. Development of dental occlusion is strongly influenced by genetic and environmental factors such as oral habits, hypertrophic tonsils and adenoids and severe chronic disease in childhood.^15^ A number of specific occlusal abnormalities in craniofacial skeletal dimensions occur in children who breathe through their mouths.^16^ Timely intervention through removal of tonsils or adenoids and other therapies have been shown to help in the treatment of these abnormalities.^17^ Adenotonsillectomy is very effective in treating OSA.^18^ The cure rates after adenotonsillectomy have been reported to range from 75-100% in normal healthy children.^19^ OSA children had posteriorly inclined mandible and increased anterior face height.^20^,^21^ In addition long-face syndrome has been described as one of the features of patients with OSA and may have a major negative impact on facial aesthetics and can be difficult to correct.^3^,^17^ In the present study no statistically significant difference was recorded between study and control group in relation to facial height, facial profile, facial or dental midlines. Most of the children in both study and control group had normocephalic facial morphology and convex facial profile, with the long-face morphology recorded in 16.7% of the study and 10% of the control group. This could be explained by the fact that low tongue posture and elongation of lower anterior facial height are apparent at three years of age but more commonly detected after age five^16^ as majority of our subjects were 5 years and younger. A comparable result regarding the facial height was reported in a group of pediatric orthodontic patients.^11^ Other studies used cephalometric radiographs reported a longer lower facial height in OSA patients as compared to control.^1^,^5^

In the present study OSA children showed significantly steeper mandibular plane angle as compared to control. Similarly several studies have reported a high mandibular plane angle in OSA subjects as compared to healthy control.^11^,^20^,^21^ Another study reported a decrease (improvement) of the mandibular plane angle of OSA children at the 5-year follow-up after adenotonsillectomy and attributed this to the greater posterior face height growth that was affected by the improved level of growth hormone after adenotonsillectomy.^2^ In the same study anterior facial height of the OSA subjects remained greater than the control subjects at the 5-year follow-up, but it increased on average by a comparable amount in both groups of children after adenotonsillectomy.^2^ Other variables evaluated in the present study and showed no significant difference between the two groups included anterior open bite, tongue size, overjet and molar relationship. A significantly increased overjet, a reduced overbite and a more anterior open bite have been reported in OSA previously.^2^ Similarly posterior crossbite showed no significant difference although it has a tendency to be more in the study group (p=0.063). The recorded posterior crossbite in the study group (23.3%) is more than that recorded for normal children in some previous studies.^22^ A high prevalence of posterior crossbite in obstructed children has been reported by other investigators.^9^,^22^

Narrower maxilla or deeper palatal height has been recorded in obstructed patients in the present study and several other studies.^2^,^9^,^23^ Some previous reports have found an increase (improvement) in maxillary arch width and spontaneous normalization of some posterior crossbites after adenotonsillectomy.^23^,^24^

In primary dentition molar relationships do not give exact expression for future relations of the dental arches, therefore it is more valid to use the canine relations when judging the sagittal relationships in the primary dentition.^15^ In this study, the study group had more class III canine relation compared to control group. In addition, the study group had fewer spaces between teeth in both maxillary and mandibular arches compared to control. A tendency for increasing maxillary and mandibular crowding was reported in the OSA children when compared with the control children.^2^ It is important that OSA in young children is diagnosed early to improve the overall health and that the patients are evaluated both from a medical and dentofacial point of view.^23^ Pediatric dentist should be aware of features of OSA and able to screen for OSA in the dental office to aid in identifying patients at risk for having OSA.

## CONCLUSIONS

Children suffering from OSA have a relatively different dentofacial morphology compared with control children. OSA subjects had deeper palatal vault, steeper mandibular plane angle and less spaced upper and lower arches compared to control.
